# Rydberg energies and transition probabilities of Li I for *np*–*ms* (*m* ≤ 5) transitions

**DOI:** 10.1186/s43088-022-00224-0

**Published:** 2022-03-26

**Authors:** R. Siddiq, M. N. Hameed, M. H. Zaheer, M. B. Khan, Z. Uddin

**Affiliations:** Department of Physics, University of Karachi, Karachi, Pakistan

**Keywords:** Rydberg atom, Rydberg levels, Transition probability, Lifetime of atoms, Lithium

## Abstract

**Background:**

Mathematical modeling provides grounds for understanding scientific systems theoretically. It serves as a guide for experimentalists in determining directions of investigation. Recently, the Covid-19 pandemic has caused disturbances in almost every walk of life. Scientists have played their role and have continued research on the effects of the pandemic. Various mathematical models have been used in different branches of science (Djilali et al. in Phys Scr 96 12 124016, 2021; Math Biosci Eng 18(6):8245–8256, 2021; Zeb et al. in Alex Eng J 61(7):5649–5665). Well-established mathematical models give results close to those obtained by experiments. The Weakest Bound Electron Potential Model is one such model, which explains hydrogen-like atoms and ions. This model has been used extensively for hydrogen-like atoms and ions to calculate energies of Rydberg levels and ionization energies. This model has been used extensively for hydrogen-like atoms and ions to calculate energies of Rydberg levels and ionization energies.

**Results:**

This paper presents the energies of the Rydberg series, 2*s*2n*s*, and 2*s*2n*p* of Li I, calculated using WBEPM. The energies are used to calculate transition probabilities from np to 2*s*, 3*s*, 4*s*, and 5*s* levels. The transition probabilities are compared with corresponding values in published data where available. The agreement with known values is good; most of the transition probabilities calculated in this work are new. A computer program was developed to find the value of the dipole matrix element. The calculations were further verified by calculating the lifetimes of some low-lying levels.

**Conclusions:**

Four series of Li I have been studied, and energies of the Rydberg levels in the series were calculated. The energies then are used to calculate transition probabilities from *np* to *ms* transitions, where *m* = 2, 3, 4, & 5 and *n* = 1–15. The results are compared where available. An excellent agreement with previously published data shows the reliability of calculations. Most of the transition probabilities are new.

## Background

Atomic lithium is less abundant in the universe because of its nuclear instability; it is a part of compounds. Lithium is an ideal atom for the spectroscopic study of astronomical data.; its 6708 Å doublet is present in K-type and cooler stars. The 6708 Å line is also among those with the maximum range of energy distribution. Furthermore, it is present in a part of the spectrum that has little effect from stellar atmospheric absorption or the crowding of lines [4]. More than 300 papers have been published on lithium describing various spectroscopic characteristics; however, only a few papers are on transition probabilities [5]. Heavens used central field approximation to calculate lifetime and transition probabilities between lower excited states of alkali atoms [6]. Fox and Dalgarno used a theoretical method and obtained eigenvalues and eigenfunctions for 1*s*^2^2*p* ^2^P^o^, 1s2*p*^2 2^P, and 1s2*p*^2 2^D states of lithium sequence. With the help of eigenfunction, they also calculated transition probabilities for these states [7]. Lingard and Nielson performed extensive calculations based on the numerical approximation method to find dipole transition probabilities, oscillator strengths, lifetimes, and branching ratios of alkali isoelectronic sequences [8]. Kostelecky and Nieto presented a potential function that reproduces spectra in the limits of quantum defect theory. With the help of this potential function, they obtained analytical wavefunctions and calculated transition probabilities of some transitions in lithium and Sodium atoms [9]. Fischer et al. presented multiconfiguration Breit-Pauli energy levels, lifetimes, and transition probabilities for the lithium sequence up to *Z* = 8 [10]. Zheng et al. employed Weakest bound electron potential model WBEPM theory to calculate transition probabilities for lithium atoms and lithium-like ions [11]. Celik used the weakest bound electron potential model and quantum defect theory to find atomic transition probabilities for the transitions in atomic lithium [12]. 2009 Wiese and Fuhr critically evaluated available literature sources and tabulated atomic transition probabilities for allowed and forbidden transitions in hydrogen, helium, and lithium [13]. Experimentally very few scientists have measured transition probabilities for the lithium atom. Most of the experimental work has measured transition probabilities of 2*p* and 3*p* levels of Li I. Anderson et al. measured lifetime and oscillator strength of 2*p* state of Li I through-beam foil excitation method [14]. Gaupp et al. also found the lifetime and oscillator strength of the 2*p* state through laser excitation in an atomic beam experiment [15]. In this work, we calculated transition probabilities of 110 transitions using the semi-empirical non-relativistic method.

## Methods

In atoms, the outermost electrons are weakly bound to the nucleus as compared to the atomic core electrons. Lithium and other atoms have a single electron in their outermost shell; this electron is the weakest bound electron. Zheng et al. developed a theory, known as Weakest Bound Electron Potential Model (WBEPM), for the spectroscopic characteristics of such atoms [16]. The weakest bound electron model theory allows us to deal with many-electron systems as a binary system; the weakest bound electron is considered an electron in a field created by a nucleus dressed up with an atomic core (consists of other electrons surrounding the nucleus). The weakest electron is excited to a higher energy level moves in an orbit that has a larger period; this reduces the coupling between core electrons and the weakest bound electron. Unlike Self consistent field (SCF) method in which each electron moves in average potential arising from other electrons, the WBEPM is based on the consideration of successive dynamic ionization [16]. The potential in which the weakest bound (WB) electron moves is given by

1 $$\begin{array}{*{20}c} {V\left( {r_{i} } \right) = \frac{A}{{r_{i} }} + \frac{B}{{r_{i}^{2} }}} \\ \end{array}$$

The radial part of the Schrodinger equation with this potential is as follows

2 $$\begin{array}{*{20}c} {\frac{{{\text{d}}^{2} R}}{{{\text{d}}r^{2} }} + \frac{2}{r}\frac{{{\text{d}}R}}{{{\text{d}}r}} + 2\left( {E - \frac{A}{{r_{i} }} - \frac{B}{{r_{i}^{2} }} - \frac{{l\left( {l + 1} \right)}}{{r_{i}^{2} }}} \right) = 0} \\ \end{array}$$

The last two terms have the same denominator to be combined. Here $A = - Z^{*}$ where $Z^{*}$ is the effective charge of the nucleus,

3 $$\begin{array}{*{20}c} {\frac{{{\text{d}}^{2} R}}{{{\text{d}}r^{2} }} + \frac{2}{r}\frac{{{\text{d}}R}}{{{\text{d}}r}} + 2\left( {E + \frac{{Z^{*} }}{{r_{i} }} - \frac{{l^{*} \left( {l^{*} + 1} \right)}}{{r_{i}^{2} }}} \right) = 0} \\ \end{array}$$

here${l}^{*}=l-{\delta }_{n} , {n}^{*}=n-{\delta }_{n}$, are effective orbital quantum number and principal quantum number, respectively, ${\delta }_{n}$ is the quantum defect in the quantum numbers and is given as a function of n.

4 $$\begin{array}{*{20}c} {\delta_{n} = a + \frac{b}{{\left( {n - \delta_{o} } \right)^{2} }} + \frac{c}{{\left( {n - \delta_{o} } \right)^{4} }} + \frac{d}{{\left( {n - \delta_{o} } \right)^{6} }}} \\ \end{array}$$

$\delta_{o}$ is the lowest quantum defect, & a, b, c, and d are found by fitting the first few energies of Rydberg levels, the energy (*E*) of the weakest bound electron is given by

5 $$\begin{array}{*{20}c} {E = - \frac{{Z^{*2} }}{{2n^{*2} }} = - \frac{{Z^{*2} }}{{2\left( {n - \delta_{n} } \right)^{2} }}} \\ \end{array}$$

The solution of Eq. (3) gives the radial wavefunction given by

6 $$\begin{array}{*{20}c} \begin{aligned} R & = \left( {\frac{{2Z^{*} }}{{n^{*} }}} \right)^{{l^{*} + \frac{3}{2}}} \sqrt {\frac{{\left( {n^{*} - l^{*} - 1} \right)!}}{{2n^{*} \Gamma \left( {n^{*} + l^{*} + 1} \right)}}} \\ & \quad \times \exp \left( { - \frac{{Z^{*} r}}{{n^{*} }}} \right)r^{{{ }l^{*} }} L_{{n^{*} - l^{*} - 1}}^{{2l^{*} + 1}} \left( {\frac{{2Z^{*} r}}{{n^{*} }}} \right) \\ \end{aligned} \\ \end{array}$$

The transition probability $(A_{fi} )$ of a transition for spontaneous emission between levels $\left( {n_{f} ,l_{f} } \right) \& \left( {n_{i} ,l_{i} } \right)$, is given by

7 $$\begin{array}{*{20}c} {A_{fi} = 20261 \times 10^{ - 6} \frac{{\left( {E_{f} - E_{i} } \right)^{3} }}{{2l_{i} + 1}}S} \\ \end{array}$$

In above expression $E_{f} > E_{i}$ and are the energies of upper and lower states, respectively, *S* represents electric dipole line strength. In lighter atoms, LS coupling dominates; therefore, line strength can be found by

8 $$\begin{array}{*{20}c} {S_{LS} = \left[ {J_{f} ,J_{i} ,L_{f} ,L_{i} } \right]\left( {\left\{ {\begin{array}{*{20}c} {L_{f} } & S & {J_{f} } \\ {J_{i} } & 1 & {L_{i} } \\ \end{array} } \right\}\left\{ {\begin{array}{*{20}c} {L_{f} } & {l_{f} } & {L_{c} } \\ 1 & {L_{i} } & {l_{i} } \\ \end{array} } \right\}P_{{l_{i} l_{f} }}^{\left( 1 \right)} } \right)^{2} } \\ \end{array}$$

The terms in the bracket contain two 6 J symbols and the matrix element $P_{{l_{i} l_{f} }}^{\left( 1 \right)}$, which is given by

9 $$\begin{array}{*{20}c} {P_{{l_{i} l_{f} }}^{\left( 1 \right)} = l_{ > } \left\langle {n_{i} ,l_{i} \left| r \right|n_{f} ,l_{f} } \right\rangle = l_{ > } \mathop \smallint \limits_{o}^{\infty } r^{3} R_{{n_{i} l_{i} }} R_{{n_{f} l_{f} }} {\text{d}}r} \\ \end{array}$$

The integral in the above expression can be found by the formula based on WBEPM theory [11, 12].

10 $$\begin{aligned} \left\langle {n_{i} ,l_{i} |r|n_{f} ,l_{f} } \right\rangle & = \mathop \smallint \limits_{o}^{\infty } r^{3} R_{{n_{i} l_{i} }} R_{{n_{f} l_{f} }} {\text{d}}r = \left( { - 1} \right)^{{n_{i} + n_{f} + l_{i} + l_{f} }} \left( {\frac{{2Z_{i}^{*} }}{{n_{i}^{*} }}} \right)^{{l_{i}^{*} }} \left( {\frac{{2Z_{f}^{*} }}{{n_{f}^{*} }}} \right)^{{l_{f}^{*} }} \left( {\frac{{Z_{f}^{*} }}{{n_{f}^{*} }} - \frac{{Z_{i}^{*} }}{{n_{i}^{*} }}} \right)^{{ - l_{f}^{*} - l_{i}^{*} - 4}} \\ & \quad \times \left[ {\frac{{n_{f}^{*4} \Gamma \left( {n_{f}^{*} + l_{f}^{*} + 1} \right)}}{{4Z_{f}^{*3} \left( {n_{f}^{*} - l_{f}^{*} - 1} \right)}}} \right]^{{ - \frac{1}{2}}} \left[ {\frac{{n_{i}^{*4} \Gamma \left( {n_{i}^{*} + l_{i}^{*} + 1} \right)}}{{4Z_{i}^{*3} \left( {n_{i}^{*} - l_{i}^{*} - 1} \right)}}} \right]^{{ - \frac{1}{2}}} \mathop \sum \limits_{{m_{1} = 0}}^{{n_{f}^{*} - l_{f}^{*} - 1}} \mathop \sum \limits_{{m_{2} = 0}}^{{n_{i}^{*} - l_{i}^{*} - 1}} \frac{{\left( { - 1} \right)^{{m_{2} }} }}{{m_{1} !m_{2} !}} \\ & \quad \left( {\frac{{Z_{f}^{*} }}{{n_{f}^{*} }} - \frac{{Z_{i}^{*} }}{{n_{i}^{*} }}} \right)^{{m_{1} + m_{2} }} \left( {\frac{{Z_{f}^{*} }}{{n_{f}^{*} }} + \frac{{Z_{i}^{*} }}{{n_{i}^{*} }}} \right)^{{ - m_{1} - m_{2} }} \Gamma \left( {l_{f}^{*} + l_{i}^{*} + m_{1} + m_{2} + 4} \right) \\ & \quad \times \begin{array}{*{20}c} {\mathop \sum \limits_{{m_{3} = 0}}^{S} \left( {\begin{array}{*{20}c} {l_{i}^{*} - l_{f}^{*} + m_{2} + 2} \\ {n_{f}^{*} - l_{f}^{*} - 1 - m_{1} - m_{3} } \\ \end{array} } \right)\left( {\begin{array}{*{20}c} {l_{f}^{*} - l_{i}^{*} + m_{1} + 2} \\ {n_{i}^{*} - l_{i}^{*} - 1 - m_{2} - m_{3} } \\ \end{array} } \right)\left( {\begin{array}{*{20}c} {l_{i}^{*} + l_{f}^{*} + m_{1} + m_{2} + m_{3} + 3} \\ {m_{3} } \\ \end{array} } \right)} \\ \end{array} \\ \end{aligned}$$

in which $$S = {\text{min}}\left( {n_{f}^{*} - l_{f}^{*} - 1 - m_{1} , n_{i}^{*} - l_{i}^{*} - 1 - m_{2} } \right).$$

## Results

Very few experimental results of transition probabilities and lifetimes are available for the transition studied in this work. In theory, only one study is found in which few transitions probabilities of lithium transitions are presented [8, 17]. Lindgren [8] used numerical coulomb approximation to calculate transition probabilities of various series for which *n* ≤ 12, l ≤ 4. The radiative transition probabilities of four Rydberg series in lithium are calculated in this work. This work employs a semi-empirical non-relativistic weakest bound electron potential model and consists of two parts. In the first part, Rydberg energies are calculated by finding corresponding quantum defects in principle quantum numbers of the Rydberg levels. The experimental values are used to calculate quantum defects and energies of the Rydberg levels and modified quantum numbers n* (Principal) and l* (orbital). The second part uses the energies and quantum defects to calculate transition probabilities for various transitions from *np* to 2*s*, 3*s*, 4*s*, and 5*s* levels. The quantum defects for Rydberg series 2*s*^2^ns and 2*s*^2^*np* are found using the following equations,

11 $$\begin{array}{*{20}c} {\delta_{n} \left( {ns} \right) = 0.39940 + \frac{0.03079}{{\left( {n - 0.412} \right)^{2} }} - \frac{0.00625}{{\left( {n - 0.412} \right)^{4} }} + \frac{0.1463}{{\left( {n - 0.412} \right)^{6} }}} \\ \end{array}$$

12 $$\begin{array}{*{20}c} {\delta_{n} \left( {np} \right) = 0.04722 - \frac{0.02292}{{\left( {n - 0.04} \right)^{2} }} - \frac{0.01225}{{\left( {n - 0.04} \right)^{4} }} + \frac{0.01734}{{\left( {n - 0.04} \right)^{6} }}} \\ \end{array}$$

These quantum defects are then used to calculate the Rydberg energies of both series. WBEPM uses Eq. (10) for the calculations of matrix element, we developed a python program that calculates dipole matrix element using wavefunction given in Eq. (9). The python program's reliability is checked by calculating the radii of various ns and *np* levels, compared with the corresponding values in the literature. An excellent agreement was found between them. A generalized formula for each ns and *np* levels radii is generated and given in Eqs. (13–14) (which is a simple quadratic equation in 'n' (principal quantum number)). The energies and the values of matrix elements are used to calculate transition probabilities for the transitions from series *np* to state 2*s*, 3*s*, 4*s*, and 5*s* up to *n* = 30. The results of transition probabilities are given in Tables 1, 2, 3, and 4. The transition probability results are compared with the corresponding values listed in NIST line data [5] and previously published results [6, 8–12]. The experimental and theoretical data of transition probabilities is available only for a few transitions, especially for transitions from lower levels; therefore, most of the results are new. The calculation of transition probabilities using WBEPM requires modified values of principal and orbital quantum numbers; the modified values of n^*^ and l^*^ are determined by the quantum defects calculated in the first part. The transition probabilities are calculated using Eq. (7). The radii of ns and *np* series can be calculated by the simple quadratic equations given below:

13 $$\begin{array}{*{20}c} { r_{ns} = 1.5n^{2} - 1.1961n + 0.2079} \\ \end{array}$$

14 $$\begin{array}{*{20}c} { r_{np} = 1.5n^{2} - 0.1517n - 0.8691} \\ \end{array}$$

**Table 1:** Transition probabilities for the transitions 1*s*^2^ *np*–1*s*^2^ 2*s* calculated in this work compared with other results

Configuration		Energy	Transition probabilities	
Upper level	Lower level	cm^−1^	This work	Other results
1*s*^2^ 2*p*	1*s*^2^ 2*s*	14,903.66	3.64E+07	3.66E+07^a^,3.69E+7^b^, 3.79E+07^c^, 3.86E+07^d^, 3.67E+07^e^, 3.27E+07^f^, 3.67E+07^g^
1*s*^2^ 3*p*	1*s*^2^ 2*s*	30,925.38	1.00E+06	8.98E+05^a^, 1.00E+06^b^, 1.20E+06^c^, 1.00E+06^d^,5.98E+05^e^, 1.7E+06^f^, 1.09E+06^g^
1*s*^2^ 4*p*	1*s*^2^ 2*s*	36,469.55	1.27E+06	1.17E+06^a^, 1.25E+06^b^, 8.48E+05^c^, 1.69E+06^f^, 1.4E+06^g^
1*s*^2^ 5*p*	1*s*^2^ 2*s*	39,015.56	8.72E+05	8.04E+05,9.00E+05, 5.85E+05
1*s*^2^ 6*p*	1*s*^2^ 2*s*	40,390.84	5.77E+05	5.31E+05^a^, 5.73E+05^b^, 3.86E+05^e^, 3.32E+06^g^
1*s*^2^ 7*p*	1*s*^2^ 2*s*	41,217.05	3.91E+05	3.59E+05^a^, 3.82E+05^b^
1*s*^2^ 8p	1*s*^2^ 2*s*	41,751.86	2.74E+05	2.59E+05^a^, 2.66E+05^b^
1*s*^2^ 9*p*	1*s*^2^ 2*s*	42,117.78	1.98E+05	1.80E+05^a^, 1.917E+5^b^
1*s*^2^ 10*p*	1*s*^2^ 2*s*	42,379.09	1.48E+05	1.38E+05^a^, 2.09E+5^b^
1*s*^2^ 11*p*	1*s*^2^ 2*s*	42,572.16	1.12E+05	1.42E+05^a^, 1.65E+05^b^
1*s*^2^ 12*p*	1*s*^2^ 2*s*	42,718.85	8.75E+04	7.93E+04^a^, 1.34E+05^b^
1*s*^2^ 13*p*	1*s*^2^ 2*s*	42,832.9	6.94E+04	1.09E+05^b^
1*s*^2^ 14*p*	1*s*^2^ 2*s*	42,923.31	5.59E+04	
1*s*^2^ 15*p*	1*s*^2^ 2*s*	42,996.21	4.57E+04	
1*s*^2^ 16*p*	1*s*^2^ 2*s*	43,055.82	3.78E+04	
1*s*^2^ 17*p*	1*s*^2^ 2*s*	43,105.21	1.09E+04	
1*s*^2^ 18*p*	1*s*^2^ 2*s*	43,146.57	2.67E+04	
1*s*^2^ 19*p*	1*s*^2^ 2*s*	43,181.56	2.27E+04	
1*s*^2^ 20*p*	1*s*^2^ 2*s*	43,211.42	1.95E+04	
1*s*^2^ 21*p*	1*s*^2^ 2*s*	43,237.11	1.69E+04	
1*s*^2^ 22*p*	1*s*^2^ 2*s*	43,259.37	1.47E+04	
1*s*^2^ 23*p*	1*s*^2^ 2*s*	43,278.78	1.29E+04	
1*s*^2^ 24*p*	1*s*^2^ 2*s*	43,295.82	1.14E+04	
1*s*^2^ 25*p*	1*s*^2^ 2*s*	43,310.84	1.01E+04	
1*s*^2^ 26*p*	1*s*^2^ 2*s*	43,324.17	8.95E+03	
1*s*^2^ 27*p*	1*s*^2^ 2*s*	43,336.03	8.00E+03	
1*s*^2^ 28*p*	1*s*^2^ 2*s*	43,346.65	7.18E+03	
1*s*^2^ 29*p*	1*s*^2^ 2*s*	43,356.19	6.46E+03	
1*s*^2^ 30*p*	1*s*^2^ 2*s*	43,364.78	5.84E+03	

^a^Lingard [8], ^b^NIST [5], ^c,d^Celik [12], ^e^Zheng [11], ^f^Heavens[6], ^g^Kostelecky[9]

**Table 2:** Transition probabilities for the transitions 1*s*^2^ *np*–1*s*^2^ 3*s* calculated in this work compared with other results

Configuration		Energy	Transition probabilities	
Upper level	Lower level	cm^−1^	This work	Other results
1*s*^2^ 3*p*	1*s*^2^ 3*s*	30,925.38	3.49E+06	3.72E+06^a^, 3.74E+06^b^. 3.78E+06^c^, 3.81E+06^d^, 3.72E+07^e^, 3.56E+06^f^, 3.7E+06^g^
1*s*^2^ 4*p*	1*s*^2^ 3*s*	36,469.55	1.55E+03	6.73E+02^a^, 6.90E+02^b^, 6.85E+02^e^, 9.07E-03^f^, 3E+04^g^
1*s*^2^ 5*p*	1*s*^2^ 3*s*	39,015.56	4.09E+04	3.87E+04^a^, 4.04E+04^b^, 2.82E+04^e^, 5.4E+04^g^
1*s*^2^ 6*p*	1*s*^2^ 3*s*	40,390.84	4.40E+04	4.23E+04^a^, 4.38E+04^b^, 3.26E+04^e^
1*s*^2^ 7*p*	1*s*^2^ 3*s*	41,217.045	3.62E+04	3.48E+04^a^, 3.61E+4^b^
1*s*^2^ 8*p*	1*s*^2^ 3*s*	41,751.86	2.80E+04	2.84E+04^a^, 2.79E+04^b^
1*s*^2^ 9*p*	1*s*^2^ 3*s*	42,117.776	2.16E+04	2.05E+04^a^
1*s*^2^ 10*p*	1*s*^2^ 3*s*	42,379.085	1.67E+04	1.66E+04^a^
1*s*^2^ 11*p*	1*s*^2^ 3*s*	42,572.163	1.31E+04	2.02E+04^a^
1*s*^2^ 12*p*	1*s*^2^ 3*s*	42,718.849	1.04E+04	9.85E+03^a^
1*s*^2^ 13*p*	1*s*^2^ 3*s*	42,832.896	8.38E+03	
1*s*^2^ 14*p*	1*s*^2^ 3*s*	42,923.314	6.83E+03	
1*s*^2^ 15*p*	1*s*^2^ 3*s*	42,996.206	5.63E+03	
1*s*^2^ 16*p*	1*s*^2^ 3*s*	43,055.825	4.70E+03	
1*s*^2^ 17*p*	1*s*^2^ 3*s*	43,105.208	2.07E+01	
1*s*^2^ 18*p*	1*s*^2^ 3*s*	43,146.571	3.36E+03	
1*s*^2^ 19*p*	1*s*^2^ 3*s*	43,181.561	2.87E+03	
1*s*^2^ 20*p*	1*s*^2^ 3*s*	43,211.423	2.48E+03	
1*s*^2^ 21*p*	1*s*^2^ 3*s*	43,237.112	2.15E+03	
1*s*^2^ 22*p*	1*s*^2^ 3*s*	43,259.371	1.88E+03	
1*s*^2^ 23*p*	1*s*^2^ 3*s*	43,278.785	1.65E+03	
1*s*^2^ 24*p*	1*s*^2^ 3*s*	43,295.818	1.46E+03	
1*s*^2^ 25*p*	1*s*^2^ 3*s*	43,310.844	1.29E+03	
1*s*^2^ 26*p*	1*s*^2^ 3*s*	43,324.167	1.15E+03	
1*s*^2^ 27*p*	1*s*^2^ 3*s*	43,336.034	1.03E+03	
1*s*^2^ 28*p*	1*s*^2^ 3*s*	43,346.651	9.25E+02	
1*s*^2^ 29*p*	1*s*^2^ 3*s*	43,356.187	8.34E+02	
1*s*^2^ 30*p*	1*s*^2^ 3*s*	43,364.784	7.55E+02	

^a^Lingard [8], ^b^NIST [5], ^c,d^Celik [12], ^e^Zheng [11], ^f^Heavens [6], ^g^Kostelecky[9]

**Table 3:** Transition probabilities for the transitions 1*s*^2^ *np*–1*s*^2^ 4*s* calculated in this work compared with other results

Configuration		Energy	Transition probabilities	
Upper level	Lower level	Energy cm^−1^	This work	Other results
1*s*^2^ 4*p*	1*s*^2^ 4*s*	36,469.55	7.38E+05	7.74E+05^a^,7.76E+05^b^, 8.04E+05^c^,7.85E+05^d^,7.73E+05^e^, 7.52E+05^f^, 7.7E+05^g^
1*s*^2^ 5*p*	1*s*^2^ 4*s*	39,015.56	2.68E+03	3.59E+03^a^, 3.39E+03^b^,4.51E+03^e^, 1.9E+03^g^
1*s*^2^ 6*p*	1*s*^2^ 4*s*	40,390.84	2.21E+03	1.62E+03^a^,1.87E+03^b^, 1.17E+03^e^
1*s*^2^ 7*p*	1*s*^2^ 4*s*	41,217.05	4.53E+03	3.69E+03^a^, 4.16E+03^b^
1*s*^2^ 8*p*	1*s*^2^ 4*s*	41,751.86	4.72E+03	4.26E+03^a^, 4.4E+03^b^
1*s*^2^ 9*p*	1*s*^2^ 4*s*	42,117.78	4.18E+03	3.43E+03^a^
1*s*^2^ 10*p*	1*s*^2^ 4*s*	42,379.09	3.52E+03	3.09E+03^a^
1*s*^2^ 11*p*	1*s*^2^ 4*s*	42,572.16	2.91E+03	4.83E+03^a^
1*s*^2^ 12*p*	1*s*^2^ 4*s*	42,718.85	2.40E+03	1.96E+03
1*s*^2^ 13*p*	1*s*^2^ 4*s*	42,832.90	1.98E+03	
1*s*^2^ 14*p*	1*s*^2^ 4*s*	42,923.31	1.65E+03	
1*s*^2^ 15*p*	1*s*^2^ 4*s*	42,996.21	1.38E + 03	
1*s*^2^ 16*p*	1*s*^2^ 4*s*	43,055.82	1.16E+03	
1*s*^2^ 17*p*	1*s*^2^ 4*s*	43,105.21	3.89E+02	
1*s*^2^ 18*p*	1*s*^2^ 4*s*	43,146.57	8.46E+02	
1*s*^2^ 19*p*	1*s*^2^ 4*s*	43,181.56	7.29E+02	
1*s*^2^ 20p	1*s*^2^ 4*s*	43,211.42	6.32E+02	
1*s*^2^ 21*p*	1*s*^2^ 4*s*	43,237.11	5.51E+02	
1*s*^2^ 22*p*	1*s*^2^ 4*s*	43,259.37	4.83E+02	
1*s*^2^ 23*p*	1*s*^2^ 4*s*	43,278.78	4.26E+02	
1*s*^2^ 24*p*	1*s*^2^ 4*s*	43,295.82	3.77E+02	
1*s*^2^ 25*p*	1*s*^2^ 4*s*	43,310.84	3.35E+02	
1*s*^2^ 26*p*	1*s*^2^ 4*s*	43,324.17	2.99E+02	
1*s*^2^ 27*p*	1*s*^2^ 4*s*	43,336.03	2.68E+02	
1*s*^2^ 28*p*	1*s*^2^ 4*s*	43,346.65	2.42E+02	
1*s*^2^ 29*p*	1*s*^2^ 4*s*	43,356.19	2.18E+02	
1*s*^2^ 30*p*	1*s*^2^ 4*s*	43,364.78	1.98E+02	

^a^Lingard [8], ^b^NIST [5], ^c,d^Celik [12], ^e^Zheng [11], ^f^Heavens [6], ^g^Kostelecky[9]

**Table 4:** Transition probabilities for the transitions 1*s*^2^ *np*–1*s*^2^ 5*s* calculated in this work compared with other results

Configuration		Energy	Transition Probabilities	
Upper level	Lower level	cm^−1^	This work	Other results
1*s*^2^ 5*p*	1*s*^2^ 5*s*	39,015.56	2.16E+05	2.34E+05^a^, 2.34E+05^b^, 2.34E+05^e^, 2.4E+05^g^
1*s*^2^ 6*p*	1*s*^2^ 5*s*	40,390.84	2.72E+03	3.65E+03^a^, 3.33 W + 03^b^, 3.71E+03^e^
1*s*^2^ 7*p*	1*s*^2^ 5*s*	41,217.05	5.67E+01	2.91E+00^a^
1*s*^2^ 8*p*	1*s*^2^ 5*s*	41,751.86	5.82E+02	5.05E+02^a^, 4.63E+02^b^
1*s*^2^ 9*p*	1*s*^2^ 5*s*	42,117.78	8.25E+02	6.88E+02^a^
1*s*^2^ 10*p*	1*s*^2^ 5*s*	42,379.09	8.50E+02	8.40E+02^a^
1*s*^2^ 11*p*	1*s*^2^ 5*s*	42,572.16	7.85E+02	1.82E+03^a^
1*s*^2^ 12*p*	1*s*^2^ 5*s*	42,718.85	6.93E+02	6.65E+02^a^
1*s*^2^ 13*p*	1*s*^2^ 5*s*	42,832.90	6.00E+02	
1*s*^2^ 14*p*	1*s*^2^ 5*s*	42,923.31	5.16E+02	
1*s*^2^ 15*p*	1*s*^2^ 5*s*	42,996.21	4.43E+02	
1*s*^2^ 16*p*	1*s*^2^ 5*s*	43,055.82	3.80E+02	
1*s*^2^ 17*p*	1*s*^2^ 5*s*	43,105.21	8.35E+02	
1*s*^2^ 18*p*	1*s*^2^ 5*s*	43,146.57	2.84E+02	
1*s*^2^ 19*p*	1*s*^2^ 5*s*	43,181.56	2.47E+02	
1*s*^2^ 20*p*	1*s*^2^ 5*s*	43,211.42	2.16E+02	
1*s*^2^ 21*p*	1*s*^2^ 5*s*	43,237.11	1.90E+02	
1*s*^2^ 22*p*	1*s*^2^ 5*s*	43,259.37	1.67E+02	
1*s*^2^ 23*p*	1*s*^2^ 5*s*	43,278.78	1.48E+02	
1*s*^2^ 24*p*	1*s*^2^ 5*s*	43,295.82	1.32E+02	
1*s*^2^ 25*p*	1*s*^2^ 5*s*	43,310.84	1.17E+02	
1*s*^2^ 26*p*	1*s*^2^ 5*s*	43,324.17	1.05E+02	
1*s*^2^ 27*p*	1*s*^2^ 5*s*	43,336.03	9.46E+01	
1*s*^2^ 28*p*	1*s*^2^ 5*s*	43,346.65	8.54E+01	
1*s*^2^ 29*p*	1*s*^2^ 5*s*	43,356.19	7.73E+01	
1*s*^2^ 30*p*	1*s*^2^ 5*s*	43,364.78	7.01E+01	

^a^Lingard [8], ^b^NIST [5], ^c,d^Celik [12], ^e^Zheng [11], ^f^Heavens [6], ^g^Kostelecky [9]

### Description of tables

The calculated transition probabilities for transitions *np* to 2*s*, 3*s*, 4*s*, 5*s* are shown in Tables 1, 2, 3, and 4. The first and second columns show the configurations of upper and lower levels of the transition, respectively. The third column shows the energies of the 1*s*^2^ *np* series calculated using quantum defect theory. The transition probabilities calculated using WBEPM for the transitions mentioned above are in the fourth column. In column five, corresponding probabilities published by other scientists are given.

#### Transitions 1***s***^2^***np***–1***s***^2^ 2***s***

Table 1 gives the transition probabilities for the transitions 1*s*^2^ *np* to 1*s*^2^ 2*s* up to *n* = 30. Our transition probability results are close to corresponding NIST values; the average difference between is 3.6%, whereas the same difference between Lingard and NIST values is 6.3%. The results of this work are closer to NIST data compared to Zheng's results [11].

#### Transitions 1***s***^2^***np***–1***s***^2^ 3***s***

Table 2 gives the transition probabilities for transitions 1*s*^2^ *np*–1*s*^2^ 3*s*. The results of this work are in good agreement with the NIST values, except for one transition probability for 1*s*^2^ 4*p*–1*s*^2^ 3*s*. Due to Cooper's minimum effect expected a large difference between the two values. Zheng’s results [11] for the transitions 1*s*^2^ 5*p*–1*s*^2^ 3*s* and 1*s*^2^ 6*p*–1*s*^2^ 3*s* have large deviations.

#### Transitions 1***s***^2^***np***–1***s***^2^ 4***s***

Table 3 gives the transition probabilities for transitions 1*s*^2^ *np*–1*s*^2^ 4*s*. The NIST values for transition probability have uncertainties up to 10% in all transitions except 1*s*^2^ 4*p*–1*s*^2^ 4*s*. Only two transition results have large deviations from published data (1*s*^2^ 5*p*–1*s*^2^ 4*s* and 1*s*^2^ 6*p*–1*s*^2^ 4*s*). The transition probabilities for the transitions 1*s*^2^ 7*p*–1*s*^2^ 4*s* and 1*s*^2^ 8*p* to 1*s*^2^ 4*s* are closer to NIST data.

#### Transitions 1***s***^2^***np***–1***s***^2^ 5***s***

Table 4 gives the transition probabilities for transitions 1*s*^2^ *np*–1*s*^2^ 5*s*. The transition probability for the transition 1*s*^2^ 6*p*–1*s*^2^ 5*s*, calculated in this work, differs from NIST values by 18%. The other values are in the acceptance range. Zheng presented transition probabilities for only two transitions; their results also agree with NIST data.

## Discussion

The possible lifetimes of Rydberg levels are determined by using the relation ${\tau }_{i}={1 \mathord{\left/ {\vphantom {1 {\sum\nolimits_{j}A}_{ji}}} \right. \kern-\nulldelimiterspace} {\sum\nolimits_{j}A}_{ji}}$ to check the reliability of the results. The lifetimes of 2*p*, 3*s*, 3*p*, and 4*p* Rydberg levels are determined and are shown in Table 5. The calculated values of lifetimes are compared with the experimental and theoretical results published in [6, 8, 17, 20–25]. An acceptable agreement is found between the lifetimes calculated in this work and the previously published results.

**Table 5:** Lifetimes of Li I's 2*p*, 3*s*, 3*p*, and 4*p* states

Level	Energy	Lifetime (ns)				
	cm^−1^	This work	Experimental value and method		Theoretical value and method	
2*p*	14,904	27.400	27.102	Photoassociation [18]	27.2	Central field approximation [6]
			27.29	Laser excited atoms [15]	27.24	Realistic potentials [17]
			27.22 (0.2)	Delayed coincidence technique [20]	27.32	Coulomb approximation [8]
			26.99 (0.16)	Photoassociation in a magneto-optical trap [21]	27.12	CI-Hylleraas method [25]
			27.11 (6)	Beam-gas-laser spectroscopy [22]	27.13	Coulomb approximation [26, 27]
			27.9 (1)	Time resolved detection [23]		
			26.9 (8)	Pulsed dye laser excitation [24]		
			26.1 (1.0)	Beam foil excitation [14]		
3*s*	27,206.1	30.1736	29.72 (17)	Beam-gas-laser spectroscopy [22]	30.46	Coulomb approximation [26, 27]
					30	Central field approximation [6]
					30.02	Realistic potentials [14]
					30.32	Coulomb approximation [8]
3*p*	30,925.4	215.578	203 (8)	Level-crossing spectroscopy [28]	209	Central field approximation [6]
			182 (6)	Level crossing spectroscopy [28]	212	Realistic potentials [17]
					216.4	Coulomb approximation [8]
4*p*	36,469.6	416.997			364	Central field approximation [6]
					383.9	Realistic potentials [17]
					402.6	Coulomb approximation [8]

**Fig. 1:**
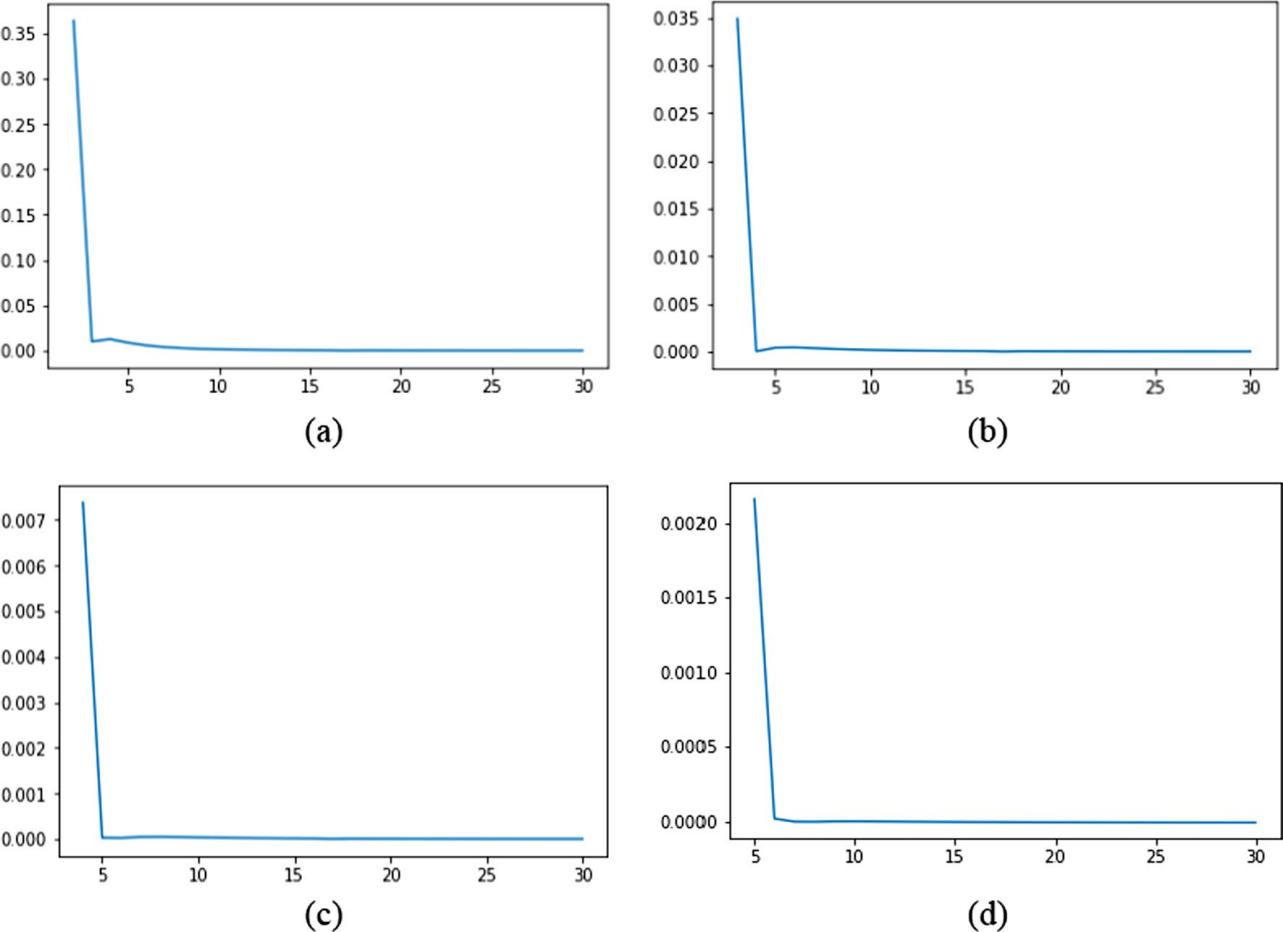
Transition probabilities as a function of effective principal quantum numbers for the transitions (**a**) *np*–2*s*, (**b**) np–3*s*, (**c**) np–4*s*, and (**d**) np–5*s*. All figures have the same horizontal axis (n^*^) and vertical axis (transition probability (10^8^))

The dipole matrix elements are zero or have a minimum value for a particular set(s) of principal quantum numbers of upper and lower levels. It occurs due to the overlapping of positive and negative amplitudes of the wavefunctions of these levels. This situation is referred to as Cooper minimum. Due to this effect, the results of transition probabilities are not in good agreement for some of the transitions. Figure 1a–d show transition probabilities for the transition *np*–*ms* (*m* = 2, 3, 4, 5), respectively. A sharp drop can be seen in the transition probabilities in all figures. It shows that the probability of transitioning to neighboring levels is higher than those with larger principal quantum numbers. As principal quantum number increases, the value of transition probability decreases slower than the initial few levels. It results in large lifetimes for higher energy levels and occurs because the transition probability depends on oscillator strength. According to Fano and Cooper [19], the oscillator strength as a function of effective principal quantum number (n*) drops extremely rapidly towards a minimum, where a reversal of sign in the R integral occurs; this contributes to most of the strength. The rise from the minimum to the second maximum for higher n* is relatively slow. The second maximum has low oscillator strength and is usually not visible in the complete spectrum.

## Conclusions

Lithium is a lighter atom, so a non-relativistic approach is suitable to calculate various spectroscopic properties of such atoms. Weakest bound electron potential theory (a semi-empirical method was employed in this work to calculate transition probabilities of various transitions from series 1*s*^2^ *np*–1*s*^2^ *ms* ($2\le n\le 30, 2\le m\le 5$). We present 110 transition probabilities, while Heavens [6], Lingard and Nelson, Kostelecky & Nieto [9], Fischer [10], Zheng et al. [11], and Celik reported 14, 48, 6, 1, 15, 4, and 35 transition probabilities, respectively. Meanwhile, NIST data shows 26 transition probabilities of these series. The results of this work are in good agreement except for four (1*s*^2^ 4*p*–1*s*^2^ 3*s*, 1*s*^2^ 5*p*–1*s*^2^ 4*s*, 1*s*^2^ 6*p*–1*s*^2^ 4*s*, 1*s*^2^ 6*p*–1*s*^2^ 5*s*) of the 110 transitions, and most of the results are new. Most of the results of transition probabilities in the literature focus on measurement of transition from 2*p* to 2*s* (experimental), and very few results of transition probabilities of lithium are reported yet. Lindgard calculated transition probabilities (theoretically) for some series of transitions in lithium for *n* ≤ 12. we extended the calculation up to *n* ≤ 30. These values of transition probabilities are helpful in the measurement of oscillator strengths and polarizabilities of Li I.

The graphs of transition probabilities show a sharp decrease towards a minimum, followed by a slight rise and eventually a slight decrease. It was observed that the dipole matrix elements have negative values for a few lower transitions that affect oscillator strengths and transition probabilities. It causes transition probabilities to have a minimum known as Cooper minimum. Cooper's minimum effect was first observed in a study of photoionization cross-section of alkali atoms. The calculations are based on the weakest bound electron potential model, which treats lithium as hydrogen-like, but hydrogen does not show zero dipole moment in photoionization. The Cooper minimum reminds us that Weakest bound electron potential model is an approximation; even though lithium is approximately hydrogen-like, it has two additional electrons in the inner shell.

Quantum defect theory was used to calculate quantum defects in principal quantum numbers, which are then used to calculate energies of Rydberg series 1*s*^2^ *np*, and 1*s*^2^ *ns*. A generalized formula of quantum defect for each of the series is developed. A computer program in python was developed and used to find the values of the matrix element; this part of the calculation makes our method slightly different from WBEPM theory. A quadratic equation can calculate the radii of the levels in ns and *np* series in 'n', which was developed to check the program's reliability. The matrix element, the energies, and quantum defects are used to calculate transition probabilities. For further verification of the results of this work, the lifetimes of some of the Rydberg levels are determined by using calculated transition probabilities. The results are compared with the previously published data, and a good agreement between the two results indicates the reliability of this work.

## Data Availability

Not applicable.

## Abbreviations

Li: Lithium
SCF: Self consistent field
WB: Weakest bound
WBEPM: Weakest bound electron potential model
